# Protolytic Reactions at Electrified TiO_2_ P25 Interface: Quantitative and Thermodynamic Characterization

**DOI:** 10.3390/molecules30030696

**Published:** 2025-02-05

**Authors:** Etelka Tombácz, Dániel Nesztor, Márta Szekeres, Hans Lewandowski, Erwin Klumpp, Renáta Gerencsér-Berta

**Affiliations:** 1 Soós Ernő Research and Development Center, University of Pannonia, Zrínyi u. 18., H-8800 Nagykanizsa, Hungary; 2 Department of Physical Chemistry and Materials Science, University of Szeged, Rerrich Béla tér 1, H-6720 Szeged, Hungary; 3 Institute of Bio- and Geosciences, Agrosphere (IBG-3), Forschungszentrum Jülich GmbH, Wilhelm-Johnen-Straße, 52425 Jülich, Germanye.klumpp@fz-juelich.de (E.K.)

**Keywords:** titania, TiO_2_ P25, surface charging, surface complexation model (SCM), calorimetry, potentiometry, partial molar enthalpy, standard enthalpy

## Abstract

Protolytic reactions on the surface of a titania photocatalyst (TiO_2_ P25 containing chlorine impurities) were studied using potentiometric and calorimetric acid-base titration. The impurity was removed by either washing or heat treatment. The efficiency of purification was tested by chlorine (TOX) analysis and acid-base titration. Common intersection points of −0.023 and −0.021 mmol/g were obtained for the original and 400 °C heat-treated samples, which are in good agreement with the measured TOX value of 28 mmol/kg. The point of zero charge of the purified sample was determined to be 6.50. Titration data were fitted to simulate protolytic reactions during isothermal calorimetric titrations of purified titania. The evolved heat was measured, and data points were corrected with the heat of mixing and neutralization. The quantity of charged surface species formed in each step of titration was calculated using the parameters from the constant capacitance model fit. The partial molar enthalpy values of the exothermic and endothermic processes of surface protonation (ΔH_pr_, −17.47 to −16.10 kJ/mol) and deprotonation (ΔH_depr_, 32.53 to 27.08 kJ/mol) depend slightly on the ionic strength of suspensions. The average standard enthalpy of one proton transfer reaction is −23.54 ± 1.75 kJ/mol, which is consistent with the literature.

## 1. Introduction

Surface hydroxyl groups are known to play a significant role in the photocatalytic behavior of TiO_2_ nanoparticles (titania). It is believed that the •OH radicals from hole-trapping by surface hydroxyl groups (≡Ti-OH) are the primary oxidizing agents in aqueous TiO_2_ suspensions [1]. The dissociative character of H_2_O adsorption on titania, producing ≡Ti-OH sites, is strongly affected by the surface structure (crystallinity, roughness, etc.) [2,3]. Dissociation prevails at surface sites with a high degree of coordinative unsaturation [4], which even affects photocatalytic hydrogen production, as revealed in a recent article [5]. It has been shown that the photocatalytic efficiency strongly depends on pH, especially in water purification [6]. In aqueous systems, the pH is a determining parameter in the operating conditions of the photoreactors, as it affects the surface charge of catalyst particles and therefore the adsorption of pollutants, colloidal stability/aggregation and suspension transparency, as well as the position of conductance and valence bands. The role of pH is widely studied [7,8], discussed [9,10], modelled [5,6,11,12] or just mentioned in the latest literature. pH-dependent surface charges originating from the protolytic reactions on ≡Ti-OH sites generate electrified interfaces [13] on the surface of TiO_2_ particles immersed in electrolyte solutions, which fundamentally influence the distribution of ions of not only electrolytes but also dissolved organic pollutants, just to mention the most frequently used model compounds such as ionic dyes (MB, AOII), phenols, chlorophenols, etc.. Recent studies [6,9,10,14,15] emphasize that adsorption on the TiO_2_ depends on the pH and ionic strength of the medium, consistent with the charge evolution on the TiO_2_ surface.

Potentiometric acid-base, frequently referred to as surface charge titration of oxides is the most commonly used method to quantitatively characterize charge formation on the reactive sites of particle surfaces [16,17,18]. Most of the literature is from the heyday of the surface charge characterization of metal oxides, decades ago. The 2-pK model of surface charging for titania describes the protonation reaction as:

≡Ti-OH + H^+^ ⇔ ≡Ti-OH_2_^+^ K_pr_, ΔG^0^_pr_, ΔH^0^_pr_ (1)

and deprotonation as

≡Ti-OH ⇔ ≡Ti-O^−^ + H^+^   K_depr_, ΔG^0^_depr_, ΔH^0^_depr_ (2)

where K_i_ is the thermodynamic equilibrium constant, ΔG^0^_i_ is the standard Gibbs free energy and ΔH^0^_i_ is the standard enthalpy of the given surface reaction. To get the standard enthalpy change of the 1-pK protonation reaction [19] we subtract Equation (2) from Equation (1) and apply Hess’s law, a basic thermochemistry,

≡Ti-O^−^ + 2H^+^ ⇔ ≡Ti-OH_2_^+^ ΔH^0^_pr_ − ΔH^0^_depr_ (3)

and divide the resulting reaction by two, as it is written for one proton transfer, thus, we can express the 1-pK protonation reaction and its thermodynamic parameters as follows:

≡Ti-OH^1/2−^ + H^+^ ⇔ ≡Ti-OH_2_^1/2+^ K_1pK,_ ΔG^0^_1pK,_ ΔH^0^_1pK_ = (ΔH^0^_pr_ − ΔH^0^_depr_)/2 (4)

For the latter reaction, the logarithm of the equilibrium constant equals the pH_PZC_, (pH of the point of zero charge (PZC)), where oppositely charged surface sites are present in equal amounts:

(5) $$pH_{PZC}=logK_{1pK}=\frac{1}{2}log\frac{K_{pr}}{K_{depr}}$$

Quantitative characterization of these reactions needs precise proton analytics. Information on the composition of interfacial layer from potentiometric titration is indirect and is most likely affected by assumptions made during data evaluation. The only direct information obtained from the experiments is the change in the activity of H^+^-ions in the bulk solution [20]. The effect of trace of impurities and additional acid-base reactions are often neglected.

The thermodynamics of interfacial acid-base reactions in aqueous oxide suspensions can be characterized by either direct isotherm titration calorimetry (ITC) or by using temperature-dependent measurements of PZC from titration or IEP (isoelectric point) measurements from electrophoresis [21,22,23,24]. In the latter, for example, the plot of pH_PZC_ vs. 1/T is a linear function and the standard enthalpy of surface protolytic process can be calculated from the slope [21,25,26].

In the interpretation of the measured calorimetric data, however, one encounters the problem of distinguishing between the different contributions [27]. In simple cases, like surface charging due to the adsorption of potential determining ions, calorimetric experiments may be designed in an appropriate way and the measured data may be interpreted [27]. Another issue is how to account for the incorporated electrostatic effect. The enthalpy of surface charging reaction (ΔH_r_) can be separated into a “chemical” (i.e., the standard ΔH_r_^0^) and an electrostatic (ΔH_r_^elec^) contribution [24].

(6) $$∆H_{r}=∆H_{r}^{0}+∆H_{r}^{elec}$$

The electrostatic contribution is described by a Gibbs-Helmholtz relationship [23]

(7) $$∆H_{r}^{elec}=F∆zT{\left(\frac{\partial \psi_{0}}{\partial T}\right)}_{p}+F∆z\Psi_{0}$$

where *F* is the Faraday constant; Δ*z* is the change in surface charge due to the adsorption reaction; *T* is the temperature; (∂ψ_0_/∂T)_p_ is the temperature coefficient of the surface potential (*ψ*_0_) at constant pressure and *F*Δ*zψ*_0_ is the electrostatic contribution to the Gibbs energy (Δ*G^elec^*). The electrostatic enthalpy contribution can be larger or smaller than the electrostatic free energy, depending on the sign of the coefficient (*∂ψ_0_/∂T*)*_p_*.

The Gibbs energy attributed to the given surface charging reaction is defined by the standard (Δ*G*^0^) and electrostatic (Δ*G^elec^*) contributions.

(8) $$∆G_{r}=∆G_{r}^{0}+∆G_{r}^{elec}$$

The 1-pK protonation reaction can be specified as half of the difference between the 2-pK protonation and deprotonation reactions. Thus, the change in standard Gibbs energy attributed to the 1-pK protonation reaction in Equation (4) is given by:

(9) $$∆G_{1pK}^{0}=\frac{\left(∆G_{pr}^{0}-∆G_{depr}^{0}\right)}{2}$$

Using Equation (5) and knowing that ΔG^0^ = -RT lnK and ΔG^0^ = ΔH^0^ − TΔS^0^ we obtain:

(10) $$pH_{PZC}=-\frac{∆H_{1pK}^{0}}{RTln10}+\frac{∆S_{1pK}^{0}}{Rln10}$$

Rodriguez-Santiago et al. [22] use the following expression for the Gibbs energy of the 1-pK protonation reaction, assuming a constant isobaric heat capacity for protonation:

(11) $$∆G_{T_{0}}^{0}=∆G_{T_{0}}^{0}-\left(T-T_{0}\right)∆S_{T_{0}}^{0}+\left(T-T_{0}-Tln\frac{T}{T_{0}}\right)∆C_{p\;T_{0}}^{0}$$

Δ*G*^0^_*T*0_, Δ*S*^0^_*T*0_, and Δ*C*^0^_*pT*0_ are the standard Gibbs energy, entropy and isobaric heat capacity of the 1-pK protonation reaction (Equation (4)), *T*_0_ is the reference temperature (298.15 K) and *T* is the working temperature. Using the thermodynamic equations above, they deduce:

(12) $${pH}_{iep,T}=\frac{∆S_{T_{0}}^{0}-∆C_{pT_{0}}^{0}\left(1+lnT_{0}\right)}{Rln10}+\frac{-∆H_{T_{0}}^{0}+∆C_{pT_{0}}^{0}T_{0}}{RTln10}+\frac{∆C_{pT_{0}}^{0}lnT}{Rln10}$$

*pH_iep,T_* is the measured isoelectric point from electrophoresis data for a given metal oxide and is equal to pH_PZC_ if there is no specific adsorption of ions other than the potential determining H^+^ ions [13]. If the reference and working temperature are the same the above Equation (12) is equal to Equation (10). Kallay et al. [21] provide the thermodynamic equations of titania for the following reaction:

≡Ti-OH_2_^+^ ⇔ ≡Ti-O^−^ + 2H^+^ Δ_dp_H^0^ = ΔH^0^_depr_ − ΔH^0^_pr_ (13)

The resulting equation that relates the *pH_PZC_* to thermodynamic properties (T = 298.15 K) is as follows:

(14) $$pH_{PZC}=\frac{{∆}_{dp}H^{0}}{2RTln10}-\frac{{∆}_{dp}S^{0}}{2Rln10}$$

The standard enthalpy and entropy for reaction (13) differ from the ones obtained by Equation (10) because the latter corresponds to the 1-pK protonation reaction. The enthalpy change for reaction (13), obtained directly from the so-called “symmetric” calorimetric measurements [24,27], in which the electrostatic contribution to the enthalpy can be neglected, is opposite in sign and twice that of the 1-pK reaction (4). It should be noted that reaction (13) is the reverse of reaction (3).

Hall [28] describes the thermodynamic aspects of the ionization process on the surface of insoluble solids with fixed dissociable groups. He derives that the temperature dependence of potentiometric titration curves relates to well-defined, calorimetrically measurable enthalpies of protonation. It is shown that the partial molar enthalpy of protonation is independent of the surface excess concentration (∂*Γ_p_*) of potential determining ions at constant temperature (*T*), pressure (*p)*, and bulk concentration of supporting electrolyte ions (*m_i_*):

(15) $${\left({\partial ∆H}_{p}/{\partial \Gamma }_{p}\right)}_{T,p,m_{i}}=0$$

which results from temperature congruence. Hall [28] stated that the heat evolved in the direct calorimetry can be attributed almost entirely to the inner region of the electric double layer where the chemical reactions occur. There is, however, only a small contribution to the total enthalpy change of surface charging (*H − H*^0^) from the diffuse layer.

In our previous work [29], calorimetric acid-base titration of alumina (Degussa C) was performed at three different ionic strengths. We demonstrated that measured heats can only be attributed to the corresponding reactions if the experiments are carefully conducted in well-controlled systems, free from side reactions caused by impurities or sample dissolution. We found that the surface reaction of protonation was exothermic, but deprotonation was endothermic. A weak dependence of reaction enthalpies on ionic strength (a few kJ/mol) was observed, as predicted by Hall [28]. The extrapolated partial molar enthalpy values at zero ionic strength (−34 and 34.6 kJ/mol for protonation and deprotonation reactions, respectively) showed excellent agreement with the calculated standard enthalpy (34.6 ± 0.6 kJ/mol) for the 1-pK protolytic reaction of alumina.

In the 1970s, titania was one of the model materials to investigate ionizable surfaces, leading to the development of surface ionization and complexation models [16], later called surface complexation models [17,30,31]. The proton-induced surface charging of nanocrystalline anatase has been studied by potentiometric titrations and electrophoretic mobility measurements [32], however, detailed thermodynamic characterization of surface protolytic reactions on titania nanoparticles still contains unanswered questions. Gun’ko et al. [33] reported the heat of immersion for titania (0.26 kJ/m^2^, exothermic) and other fumed oxide powders. Previous work on calorimetric acid-base titrations of aqueous rutile suspensions revealed that proton adsorption and desorption enthalpies were largely reversible in respect to sign over the range of pH 4–10 and no dependence on ionic strength (in 0.01–0.1 M NaNO_3_) was apparent [23]. De Keizer and coworkers [34] measured the heat of proton adsorption for rutile using ITC and the calorimetric heat (−21 kJ/mol) at the PZC agreed well with the corresponding enthalpies derived from the shift of pH_PZC_ with temperature (ΔH_pr_ = ∂pH_PZC_/∂T = −18 kJ/mol for TiO_2_). They concluded that the enthalpy of charge formation depends on the nature of the oxide but is practically independent of surface charge density and electrolyte concentration [34]. Kallay and coworkers [21] investigated the calorimetric heat effects accompanying the acid-base titration of purified TiO_2_ P25. They determined the difference in partial molar enthalpies of protonation and deprotonation reactions (50 kJ/mol), which was similar to the value obtained from their calorimetric titration (55 ± 5 kJ/mol). In a later published study by [22], high-temperature electrophoresis data (isoelectric point (IEP) and zeta potential) of metal oxides, including TiO_2_, were used for thermodynamic analysis to derive standard enthalpies for surface protonation reactions. Rodriguez-Santiago and colleagues also used a combination of crystal chemical and solvation theories to determine these enthalpy values. The 1-pK protonation enthalpy for rutile was −23.2 and −24.7 kJ/mol [22].

The aim of our work was to characterize the pH-dependent surface charging of the most frequently used photocatalyst, TiO_2_ P25 nanoparticles, and to investigate the effect of the concentration of an indifferent electrolyte. The potentiometric and calorimetric measurements were meticulously planned and conducted under controlled experimental conditions, based on our previous experience with sample pretreatment and modeling [29]. We evaluated the results of potentiometric and calorimetric acid-base titrations, interpreting the likely surface charging reactions and the influence of the electric double layer on the partial molar enthalpy of surface protolytic processes. Additionally, we aimed to highlight the similarities and differences between our findings and those reported in the literature through comparative analysis.

## 2. Results and Discussion

### 2.1. Quantitative Characterization of Surface Charge Formation on Titania Nanoparticles

Different metal oxides may have varying impurities depending on the production process used. TiO_2_ P25, a commercial product, contains chlorine contamination as a by-product of the flame hydrolysis of TiCl_4_. In aqueous media, this chlorine content undergoes hydrolysis, releasing acidic species into the bulk liquid phase, which lowers the pH of suspension to an acidic range of 3.5–5.5 in dense suspensions. Heat treatment at high temperature has been proven effective for removing chlorine contamination from similarly produced alumina [35]. However, care must be taken when selecting the temperature, as crystalline phases like the well-known anatase-to-rutile phase transition, may undergo degradation above 400 °C [36]. An alternative purification method is exhaustive washing of the oxide sample with pure water, which is more favorable as it should preserve the crystalline structure.

#### 2.1.1. Chlorine Impurity: Measurement and Removal

The chlorine impurity of TiO_2_ P25 sample results in a total HCl content of less than ~8.2 × 10^−5^ mol/g, as calculated from the value (<0.3%) provided by the manufacturer [37]. A significantly lower concentration, 2.8 × 10^−5^ mol/g, was determined by the TOX (Total Organic Halogen) measurement for the original TiO_2_ sample. After heat treatment at 400 and 600°C, the chlorine content was reduced to 2.6 × 10^−5^ and 1.4 × 10^−6^ mol/g, respectively. Thorough washing of the TiO_2_ P25 sample—facilitated by raising pH to 10 and then lowering it—resulted in a further reduction of chlorine to 1.6 × 10^−6^ mol/g. It can be stated that both exhaustive washing of TiCl_4_ hydrolysis products in aqueous suspension and heat treatment of the titania powder at 600 °C are effective methods for chlorine removal.

#### 2.1.2. Potentiometric Titration

Potentiometric acid-base titration is one of the most frequently applied methods for quantitative characterization of surface charge formation, but one must treat experimental data with care, taking the limitations of the method into consideration. A common mistake is neglecting impurities and additional reactions, attributing all H^+^/OH^−^ consumption solely to surface charge formation. Dissolution of the given oxide is also a substantial factor when choosing the pH-range of the acid-base titration. Unlike alumina [35] or ZnO_2_, TiO_2_ can be used safely over a broader pH-range (2–12), where the dissolution of the sample may be negligible The result of the potentiometric titration of oxides is the net proton consumption vs. pH function. To consider these as specific net surface proton excess functions, they must meet the following criteria: (i) reversible up and down curves, (ii) appropriate ionic strength dependence of the curves, (iii) coincidence of the common intersection point (CIP) of the curves and theoretical PZC. If not, one must consider specific ion adsorption or the presence of acidic/basic impurities, and purification of the sample is required. If the CIP of the curves depends on the quality of the background electrolyte used and pHcip ≠ pH_PZC_, specific adsorption should be considered. If pHcip = pH_PZC_ but CIP ≠ PZC, this indicates the presence of some free acid or base. To obtain an ideal reference state, purification of the sample is necessary to achieve CIP = PZC [13,20,38].

**Figure 1:**
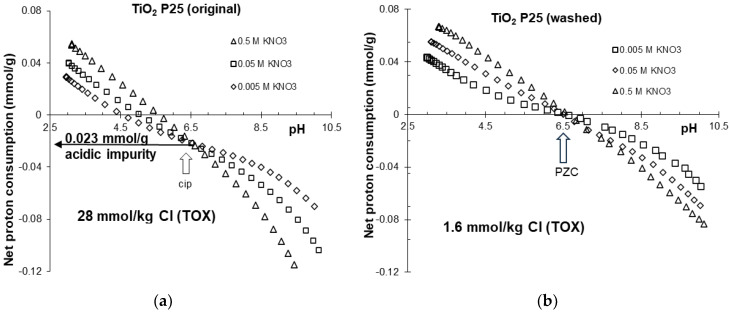
Net proton consumption vs. pH functions of the original (**a**) and washed (**b**) titanium oxide samples in 0.5, 0.05 and 0.005 M KNO_3_.

**Figure 2:**
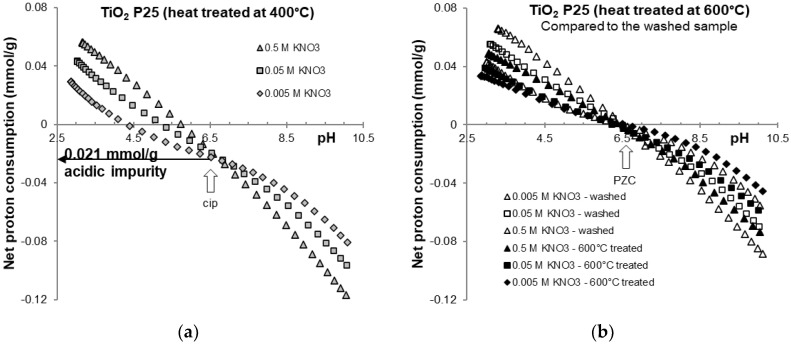
Net proton consumption vs. pH functions of the heat-treated samples of titania: on 400 °C (**a**) and 600 °C (**b**) in 0.5, 0.05 and 0.005 M KNO_3_. For comparison the curves of washed sample is also given (**b**).

Potentiometric acid-base titration of heat treated and purified samples were carried out over the range of pH 3 to 10 in the indifferent electrolyte KNO_3_. The net proton consumption vs. pH curves of the three different background electrolyte concentrations intersect at a common intersection point (CIP). The curves are shown in Figure 1 and Figure 2 and are similar to those frequently found in the literature [16,39]. Due to the chemical stability of titania in the pH region studied here, no sign of solid-phase dissolution was observed. The pH_PZC_ values determined from the net proton consumption vs. pH curves for the purified titania samples at the different ionic strengths were very similar to the values reported for TiO_2_ P25 [17], with an average value of pH = 6.5 ± 0.1.

Considering the free acid released upon the hydrolysis of TiCl_4_ in reaction (19) the observed shift (shown in Figure 1a) of the CIP to negative values of the net proton consumption for the original and purified samples is attributed to the acidic (HCl) impurity. The estimated HCl concentration of 2.25 × 10^−5^ mol/g for the original sample is similar to the value obtained from the TOX measurement (2.8 × 10^−5^ mol/g).

The net proton consumption curves of the original sample after heating at 400 °C in Figure 2a clearly demonstrate that the amount of acidic impurity does not significantly decrease. Therefore, heat treatment at this temperature was not successful in removing chlorine contaminants from the sample. Heating the sample at temperature of 600 °C resulted in shifting of the CIP of the curves to zero net proton consumption (pH_PZC_). Although the sample appears to be purified, a significant (20–30%) decrease in the amount of ≡Ti-OH active sites available for surface protolysis is observed in the Figure 2b, where titration data for the washed and heated samples are compared. This is likely a consequence of the anatase-to-rutile phase transition, which is possible at this temperature [36]. As a result, the exhaustive washing process used to purify the TiO_2_ P25 sample is more favorable compared to alumina purification, where heat treatment has been the effective method to reduce chlorine content [35]. The remaining impurity in the washed sample (1.6 × 10^−6^ mol/g by TOX) suggests that the hydrolysis process could be hindered by chloride ions already present in the equilibrium liquid phase. This amount of chlorine contaminant and the resulting hydrolysis product is too small to be detected by the proton analytics used. We are convinced that a well-designed washing procedure better preserves the active sites, as heat treatment is accompanied by surface dehydration and dehydroxylation at higher temperature, in addition to the potential phase transformation of the crystal lattice. Notably, it has been recently reported that the residual chloride impurities can affect the adsorption of target molecules but have no impact on photocatalytic performance [9].

**Figure 3:**
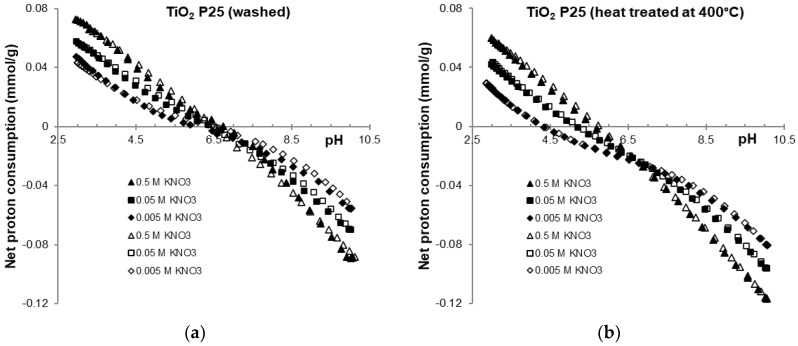
Reversibility of the titration of washed (**a**) and heat treated (**b**) titanium oxide P25 samples, up titration (open symbols, increasing pH) with KOH solution; down titration (filled symbols, decreasing pH) with HNO_3_ solution.

To check the reversibility of the processes, the forward and backward curves were compared. As shown in Figure 3, the acid-base reactions—i.e., surface charging of titania and neutralization of its impurities—are reversible at each concentration of indifferent KNO_3_ electrolyte, within the experimental error of potentiometric titrations. This type of comparison is rarely found in the literature, as previously discussed [20]. The clear separation and identification of the CIP points is also exceptional, which was made possible by our unique calibration process developed [20]. We use double calibration: both the pH scale with standard buffers and the H^+^/OH^−^ concentration with blank electrolyte titrations are calibrated. Therefore, potentiometric measurements allow for concentration-based calculations, eliminating the need for CIP shift or any other empirical correction to identify PZC of TiO_2_P25. We believe that this noteworthy achievement will attract professional interest.

#### 2.1.3. Evaluation of Titration Results: Numerical Fitting

The need to fit surface protolytic equilibria has led to the application of increasingly complex theoretical approaches in surface complexation models (diffuse double-, triple-, and four layer models) [16,30,40,41,42,43,44] and to the introduction of surface site heterogeneity parameters. While the smearing effect of the electrostatic field formed during surface protolytic processes is fundamental, the heterogeneity of proton binding sites at the oxide/solution interface has been studied both theoretically [45,46,47] and experimentally [42,43,48,49,50]. Notably, surface complexation modeling has recently been used, for example, to determine quantitative equilibrium reaction constants for nucleic acid adsorption on titanium oxide surfaces [51].

When evaluating experimental data from the acid-base titration of amphoteric solid materials, it is important to consider that other acid- and base-consuming reactions (e.g., impurities, dissolution of solid at low or high pH values) can occur in parallel with surface-charging processes, and these reactions cannot be separated experimentally.

**Figure 4:**
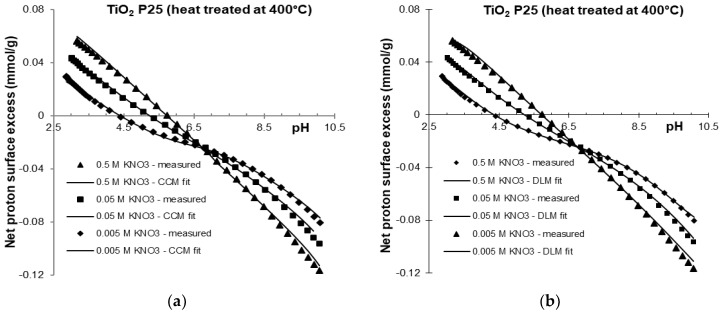
FITEQL fit of experimental data of heat treated (400 °C) titanium oxide samples in 0.5, 0.05 and 0.005 M KNO_3_ using constant capacitance (CC) (**a**) and diffuse double layer (DL) (**b**) models (solid lines).

**Figure 5:**
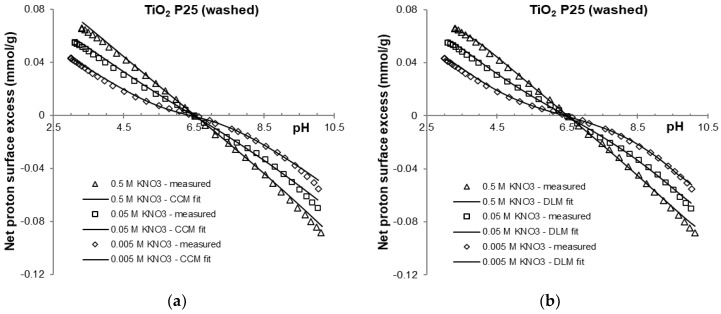
FITEQL fit of experimental data of washed titanium oxide samples in 0.5, 0.05 and 0.005 M KNO_3_ using constant capacitance (CC) (**a**) and diffuse double layer (DL) (**b**) models (solid lines).

The potentiometric acid-base titration data for the washed and 400 °C heat treated titania samples were fitted using the data-fitting program FITEQL [52]. FITEQL optimizes the chemical equilibrium constants and selected parameters to minimize the difference function between the experimental and calculated values obtained from the multi-component chemical equilibrium. To define the chemical equilibrium protonation and deprotonation reactions of the titania surface Equations (1) and (2) were used. Additionally, to account for the base consuming impurity, the hydrolysis of TiCl_4_ Equation (19) was incorporated into the equilibrium definition. Surface complexation models are also integrated into the program to account for interactions at charged interfaces. The choice between different surface complexation models—such as the constant capacitance (CC), diffuse layer (DL), Stern, and triple layer (TL) models—is optional. The experimental data from the forward titration in indifferent electrolyte (KNO_3_) solutions was well fitted using any of these surface complexation models. The experimental data and fit of the FITEQL optimization procedure using the CC and DL models are compared in Figure 4 and Figure 5.

The optimized intrinsic equilibrium constants and model parameters used for surface charging reactions (1) and (2), which occur on the surface hydroxyl groups of titania in different electrolyte solutions, are summarized in Table 1 and Table 2. Total concentration of surface sites can be treated as adjustable model parameters within reasonable limits, as can the capacitance values for the electric double layer [44]. In the FITEQL optimization procedure, the intrinsic equilibrium constants of the protonation and deprotonation reactions and the total concentration of ≡Ti-OH surface sites were fitted. Capacitance values were adjusted to achieve the best fit, ensuring reasonable values for T(≡Ti-OH) and log K. The chemical equilibrium of the surface protolytic reactions was calculated at each ionic strength (0.005, 0.05, 0.5 M KNO_3_) using the fitted parameters. The goodness of fit, expressed as WSOS/DF (weighted sum of squares/degrees of freedom), was reasonably good, with values below 20 [52]. The absolute values for intrinsic equilibrium constants (log *K*_a1_^int^, log *K*_a2_^int^) increased with increasing electrolyte concentration. At a given pH, the concentration of charged surface sites was higher at higher electrolyte concentration, indicating a stronger charge screening effect from the indifferent electrolyte. For the experimental data of the 400°C heat-treated titania, the total concentration of TiCl_4_ was fixed at the overall best-fit value of 4.18 × 10^−4^ M (~0.02 mmol/g) to account for the base consuming impurity. This value is very similar to that obtained from TOX measurements, as shown in Figure 1. The equilibrium constants and pH_PZC_ values obtained from the fit are consistent with those reported in the literature for P25 and other forms of TiO_2_ [53,54,55,56]. The fitted total concentration of surface sites varied within a reasonable range for titanium dioxide (2.7–13 sites/nm^2^) [17]. The fitted log K values for the protonation reaction of the heat-treated (4.42–4.67) and washed sample (4.47–4.57) were nearly identical, as were the values obtained for the deprotonation reaction. Surface charge titrations provide more detailed information than the advanced methods such as polarimetric angle-resolved second harmonic scattering, which only estimate *pKa* (= −*log Ka*) and PZC values by detecting changes in the orientation of water molecule at the TiO_2_ interface as a function of pH [7]. The range of optimal capacitance values of the CCM fit for both samples was also very similar (0.8–0.95 F/m^2^ for the heat-treated sample and 0.76–0.94 F/m^2^ for the washed samples). The CCM fitting parameters (76–94 μF/cm^2^) for TiO_2_P25, a mixture of non-porous anatase, rutile, and amorphous phases (in a 78:14:8 ratio, with mean particle size of 21 nm) are well aligned with experimental data from the literature. For example, surface-normalized capacitance values of 120, 110 and 130 μF/cm^2^ were reported for anatase nanoparticles with sizes of 7, 10 and 30 nm, respectively by Wang et al. [57]. It should be noted that much higher higher capacity values (e.g., 239–134 mF/cm^2^ for nanotubes) have been reported in recent literature [58]. We can conclude that experimental results from oxide titration using accurate proton analytics [20] can be successfully fitted by surface complexation models, even when the sample contains acid impurity, which can be treated as additive base consuming component. The results show that experimental data for the heat-treated sample containing acidic impurities can also be fitted, if the equilibrium is appropriately described.

The model parameters obtained from the CCM fit of the data for washed titania were used to simulate the calorimetric titration. This made it possible to attribute the measured heat evolution to specific surface reactions [29].

### 2.2. Thermodynamic Characterization of Surface Charge Formation on Titania Nanoparticles

#### 2.2.1. Calorimetric Titration

**Figure 6:**
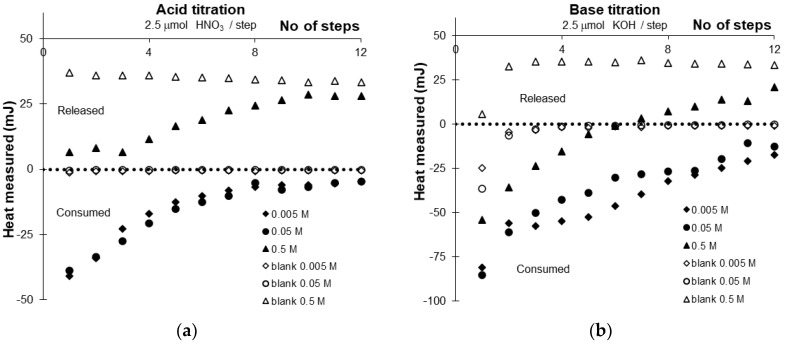
Heat measured during the calorimetric acid (**a**) and base (**b**) titration of washed titanium oxide suspensions in 0.5, 0.05 and 0.005 M KNO_3_ in parallel with blank titrations of electrolyte solutions.

During the titrations of TiO_2_ suspensions and blank electrolyte solutions with standard acid and base titrants, the heat flow was measured in the titration cell of the TAM calorimeter. The calorimeter signal peaks were used to calculate the gross amount of released or consumed heat. The heat of titration in each step was plotted as a function of the number of steps for both the acidic and alkaline series at each ionic strength, as shown in Figure 6.

Measured heat during the blank titration was not negligible, so the heat of the suspension titration was corrected at each step using the corresponding blank heat value. Our observations indicate that the heats for suspension titrations are exothermic, while the corresponding blank titrations show practically zero heat flow. The exception occurs at the highest ionic strength (0.5 M KNO_3_), where the suspension titration heat is endothermic. However, since the blank titration is even more endothermic, the corrected value after accounting for the blank data remains exothermic.

By correcting with the blank titration, the heat of mixing from Equation (21) was also subtracted. However, the cumulative heat vs. amount of added reactant (acid or base) functions were linear only for the first three steps; after that, they curved to a smaller slope. This indicated that the exact extent of the surface reactions of titania needed to be accounted for.

#### 2.2.2. Evaluation of Titration Results: Simulation of Extent of Reactions in Calorimetric Cell

A reliable model must be applied to calculate the extent of the surface reaction caused by the added amount of reactant. To calculate the enthalpy changes related to the extent of the surface reactions, we calculated the amount of charged surface species formed during the calorimetric titrations at all ionic strengths using the previously applied CCM surface complexation model.

The reference state of the aqueous TiO_2_ suspension (25 °C, pH set at the PZC of the suspension, and the presence of an indifferent 1:1 electrolyte) was fixed as the starting point. Titration with acid and base solutions was simulated using the CC model parameters obtained for the washed titania (Table 2). The equilibrium concentration of the corresponding species at each simulated step was calculated by the program FITEQL. The amount of charged surface species formed in the H^+^ and OH^−^ association reactions

≡Ti-OH + H^+^ ⇔ ≡Ti-OH_2_^+^   log K_1_^int^ (16)

≡Ti-OH + OH^−^⇔ ≡Ti-O^−^ + H_2_O log K_2_^int^ (17)

in each titration step (addition of 2.5 μmol H^+^/OH^−^) was calculated at different concentrations of 1:1 electrolyte using the CCM without optimization. The model parameters were taken from Table 2. The equilibrium concentration of H^+^ ions was calculated, with the corresponding acidic end pH values being 2.72, 2.79, and 2.88, and the alkaline end pH values being 11.04, 10.86, and 10.64 in 0.005, 0.05, and 0.5 M indifferent electrolyte, respectively. These were compared to the final pH values measured at the end of calorimetric experiments. The measured pH values at the end of acid titration were 2.57, 2.59, and 2.62, and for the base titration were 11.73, 11.99, and 11.89 in 0.005, 0.05, and 0.5 M KNO_3_ electrolyte, respectively. We used the equilibrium speciation of surface species calculated for the subsequent steps of titration in the evaluation of the calorimetric data. Due to the deviation between the measured and calculated end pH values on the alkaline side the data from CCM model simulation above pH = 10.5 were not used.

Reaction (1) is actually the surface protonation process; however, the OH^−^ association reaction Equation (17) is different from the surface deprotonation reaction given in Equation (2) (≡Ti-OH ⇔ ≡Ti-O^−^ + H^+^) because Equation (17) involves the formation of water:

H^+^ + OH^−^ ⇔ H_2_O   ΔH^0^ = −56.5 kJ/mol (18)

Therefore, the heat of water formation in each step must be subtracted from the measured heat of titration with base solution if we wish to identify the reaction heat of surface deprotonation reaction of Equation (2), i.e., the negative charge formation on the oxide surface.

**Figure 7:**
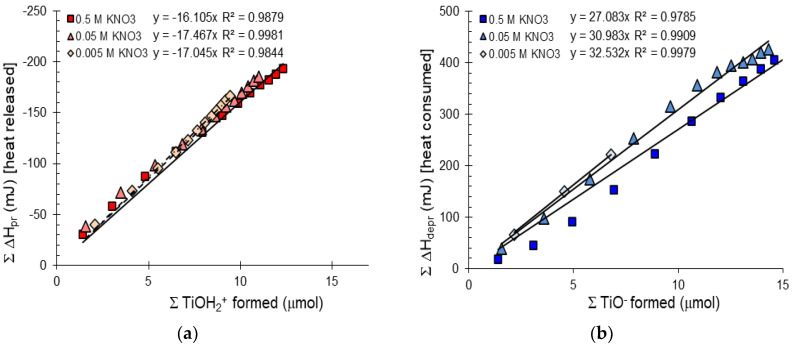
The cumulative heat of surface protonation (**a**) and deprotonation reactions (**b**) calculated with correction for water formation plotted against the calculated amount of surface charge of purified titanium oxide samples in 0.5, 0.05 and 0.005 M KNO_3_.

The cumulative heat of surface protonation and deprotonation reactions (calculated with correction for water formation) during titration was plotted as a function of the calculated amount of surface charges formed in reactions (1) and (17) (Figure 7). The plots of experimental data (Figure 7) show a satisfactory linear relationship for both the protonation and deprotonation reactions on the surface sites of titania. The linearity indicates that the molar enthalpy changes of surface charging reactions (ΔH_depr_ and ΔH_pr_) are independent of surface charge density within experimental accuracy, i.e., the temperature congruence given in Equation (15) is valid under the present experimental conditions. The occurrence of temperature congruence implies that there is no significant specific adsorption of supporting electrolyte ions, there is only one type of surface group (or the contribution of different types is equal at any pH value), and the interaction between neighboring surface groups is not influenced by the protonation or deprotonation of either [28]. Thus, the slope of the cumulative heat of surface charge formation vs. the amount of charged sites function in Figure 7 can be identified with the partial molar enthalpies of surface protonation and deprotonation reactions (ΔH_pr_ and ΔH_depr_). The calculated values are summarized in Table 3.

With increasing concentration of indifferent electrolyte, the partial molar enthalpy values of the 2-pK surface protonation and deprotonation reactions are given in Table 3. Applying Hess’s law, as previously discussed, the enthalpy for the 1-pK protonation reaction can be calculated using Equation (4). Partial molar enthalpies for the 2-pK and 1-pK reactions are summarized in Table 3. One of the surface charging models of oxides is the amphoteric site concept (2-pK concept), which involves protonation/deprotonation reactions of surface hydroxide groups as written in the first two reactions (Equations (1) and (2)). The other is the coordination concept (1-pK concept) which assumes protonation of only one kind of surface site, given in the resulting equation. We found that the calculated, 1-pK protonation enthalpy value −23.54 ± 1.75 kJ/mol is in good agreement with the standard reaction enthalpy provided by Kallay et al. [21] for TiO_2_ P25. The previously mentioned work gave molar enthalpy values of 50 kJ/mol and 55 ± 5 kJ/mol for the reaction in Equation (13) (temperature dependence of pH_PZC_ and symmetric calorimetry measurement, respectively), which should be divided by 2 to be conform with the 1-pK protonation reaction (4). The general values for metal oxides range between –20 and –50 kJ/mol [59]. We believe that our calorimetric results contribute to addressing and resolving the fundamental debate regarding 1-pK and 2-pK approaches of the protolytic reactions at electrified interface of TiO_2_.

Molar enthalpy values found in other sources also comply well with our present results (Table 3). The reversible proton adsorption and desorption enthalpy values determined for rutile by Machesky and Anderson [23] are ±14.7 and ±30.0 kJ/mol, respectively. De Keizer et al. [34] reported molar enthalpy values of −22 and −32 kJ/mol (when corrected for the neutralization reaction: 24.5 kJ/mol) for the same processes, respectively. Kallay and Ćop [60] also presented a theoretical work where numerical fitting of a model metal-oxide with the parameters (Δ*H*_pr_ = −15 kJ/mol Δ*H*_depr_ = 30 kJ/mol pH_PZC_ = 6.56) very similar to our sample was used.

**Figure 8:**
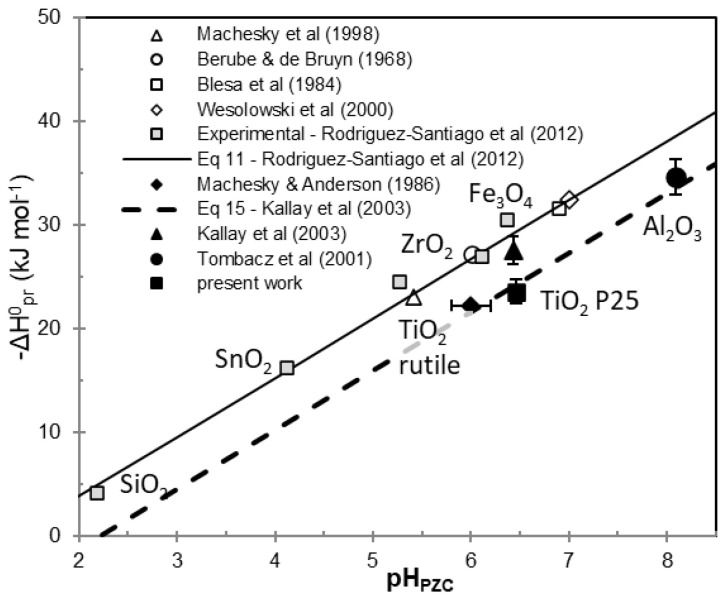
Standard enthalpies of the 1-pK protonation reaction. Open and grey symbols are the re-plot of collected and measured experimental data of [22]. Solid symbols are experimental data from additional sources [21,23,29] and the present work. The solid and broken lines represent the predictions of Equations (10) and (14), respectively.

The work of Rodriguez-Santiago et al. [22] presented standard enthalpies for the 1-pK protonation reaction. By assuming constant values for heat capacity and entropy change in Equation (12) it is possible to determine the standard protonation enthalpy knowing only the pH_PZC_ of the sample. They used constant values of ΔS = 25.5 J/mol K and ΔC_p_ = 87 J/mol K to calculate standard protonation enthalpies for titania data from literature [25,26]. The enthalpies in the latter work for standard state, determined by thermodynamic approach of the 1-pK model and the combination of crystal chemical and solvation theory, were −23.2 and −24.7 kJ/mol, respectively, for experimental data of rutile. Kallay et al. [21] applied Equation (14) and ΔS = −85 J/mol K to calculate the enthalpy for titania P25 and compared it with the result obtained from their symmetrical calorimetry experiment. We used the data available in the above articles and their referenced works and compared them with our results presented here for titania, as well as previously published data for alumina [29]. Standard enthalpies for the 1-pK protonation reaction of metal oxides are plotted against their pH_PZC_ values in Figure 8. To compare the experimental values with the theoretical predictions of Equations (10) and (14), the straight lines corresponding to these equations are also plotted in Figure 8.

We calculated the standard enthalpy of the 1-pK protonation for our purified titania using the pH_PZC_ (6.46). By applying Equation (10) with the entropy value ΔS = 25.5 J/mol K, the resulting enthalpy was approximately −29 kJ/mol. However, when Equation (14) was used with ΔS = −85 J/mol K, and the enthalpy value was converted to the 1-pK protonation reaction enthalpy, we obtained a value of around −24 kJ/mol. Interestingly, the experimental values for surface protonation enthalpy of rutile (open triangle and square) and titania P25 (black triangle and square) are nearly identical, with the only difference being their pH_PZC_ values. The experimental value for rutile (black diamond), as determined by Machesky and Anderson [23], falls between these points. The change in entropy, as chosen by Kallay et al. [24,27] in Equation (13) also seems appropriate, particularly when comparing the protonation enthalpy of alumina determined previously by IT calorimetry (Figure 8). Although the two theoretical lines in Figure 8 show a non-negligible difference, this difference becomes insignificant when considering the experimental error associated with calorimetric experiments. The thermodynamic parameters obtained pertain to the reactions occurring at ≡Ti-OH sites on an electrified interface and, therefore, should be generalizable to any TiO_2_ surface. Recent simulations have shown that the surface chemistry of TiO_2_ in aqueous environment depends on various factors beyond pH, such as the crystal structure and the morphology of the TiO_2_ particles [11]. Consequently, different equilibrium constants for protonation and deprotonation, point of zero charge values, pH-dependent speciation of charged and adsorbed species, and the pH windows for their existence have been calculated using ab initio molecular dynamics with a grand canonical formulation for different crystal faces of rutile and anatase particles. While our calorimetric measurements were performed under ultrapure conditions, the results are broadly applicable to other conditions, provided that the rule of thermodynamics are followed. Our data can, for instance, be integrated into equilibrium specification models where environmental conditions are also considered.

## 3. Materials and Methods

### 3.1. Materials

The chemicals used in this study, including KOH, KNO_3_, HNO_3_ solutions and standard buffer solutions, were analytical reagent grade and were sourced from Merck except for the titanium dioxide P25 powder. The latter was a highly dispersed commercial product from Degussa AG. This fine TiO_2_ powder is produced through the flame hydrolysis of TiCl_4_ resulting in a mixture of anatase, rutile and amorphous phases with the phase composition determined to be 78% anatase, 14% rutile, and 8% amorphous, as reported by [61]. According to the technical data sheet from Evonik-Degussa AG, the product has a specific surface area of 50 m^2^/g, a mean particle size of 21 nm, and an HCl content less than 0.3% [37]. Millipore MilliQ water was used in all experiments to ensure ultrapure conditions. It is assumed that the HCl content in the TiO_2_ P25 originates from residual, non-hydrolyzed TiCl_4_. Under appropriate condition, the hydrolysis of TiCl_4_ occurs according to the following reaction:

TiCl_4_ + 2H_2_O ⇔ TiO_2_ + 4H^+^ + 4Cl^−^ (19)

This reaction generates TiO_2_ along with hydrochloric acid (HCl), which contributes to the residual acid impurity present in the commercial product.

To eliminate chlorine contamination from the powder TiO_2_ P25 samples, two approaches were employed: heat treatment and extensive washing. In the first approach, the samples were heated in an oven at 400 or 600 °C for 6 h in air, without the need for an inert atmosphere like nitrogen (N_2_) or argon (Ar). In the second approach, the purification of the titania powder was carried out by exhaustive washing in Millipore MilliQ water. To enhance the hydrolysis of any remaining TiCl_4_ impurity, the pH of suspensions was initially raised to 10. The alkaline suspension was stirred for half an hour to promote hydrolysis. Afterward, the pH was reduced to approximately 6 to facilitate the washing process. This washing procedure was repeated three times, using ample MilliQ water, with the supernatant being separated by centrifugation after each wash. The effectiveness of the purification was monitored using TOX analysis and potentiometric measurements, which helped to ensure the successful removal of chlorine-containing impurities.

### 3.2. Methods

#### 3.2.1. TOX Determination

A coulometric analyzer (Euroglas TOC 1200) was used to measure the chlorine content of samples as total organic halides (TOX). After pretreatment, the samples were oxidized at high temperatures in a combustion furnace. During this process, combustion gases, carrying halide ions, were released and led into the titration cell, where a coulometric titration took place using silver ions (Ag^+^). The total halogen was calculated from the precipitated amount of AgX. TOX was determined for our original and purified samples of TiO_2_ P25.

(20) $$\mathrm{Oxidation}:\;R-X+O_{2}\to HX+CO_{2}+H_{2}O\;\mathrm{Titration}:\;HX+Ag^{+}\to H^{+}+AgX$$

#### 3.2.2. Potentiometric Titration

Acid-base titration was used to determine the pH-dependent surface charge state in a CO_2_-free atmosphere. We used different concentrations of background KNO_3_ electrolyte to maintain the constant ionic strength at 0.005, 0.05, and 0.5 M. Solid samples were equilibrated with the electrolyte solution for an hour, with gentle magnetic stirring under a continuous stream of purified, wet nitrogen. After this sample pretreatment, the equilibrium titration was performed using a unique self-developed system (GIMET1) with 665 Dosimat (Metrohm) burettes, nitrogen bubbling, magnetic stirrer, and high-performance potentiometer. The whole system (mV-measure, stirring, bubbling, amount and frequency of titrant) was controlled by IBM PS/1 computer using AUTOTITR software. To check Nernstian response, a Radelkis OP-0808P (Hungary) combination pH electrode was calibrated for three buffer solutions. Using the data from the reference solution titration (blank), the hydrogen ion activity vs. concentration relationship was determined so that the electrode output could be converted directly to hydrogen ion concentration instead of activity. To reach a starting pH of about 3, a calculated amount of standard HNO_3_ solution was added to the titania suspensions (50 mL, 20 g/L) equilibrated with electrolyte. To provide a CO_2_-free environment the suspensions were purged with nitrogen for 20 min. After the pre-purge phase, the samples were titrated by standard KOH solution up to pH 10, and then by standard acid solution down to pH 3. To control the addition of a new titrant dose, a criterion for the change of pH with time (ΔpH/Δt) was introduced. After addition of each titrant portion, a sampling cycle (stirring, waiting, and measuring pH) was repeated at least three times. To maintain a steady increment in pH, the amount of subsequent portions is estimated from the last three measured points. Optimal parameters for equilibrium titration were as follows: the criterion for pH settling is 0.0002 pH/s; the sampling cycle is 15 s; the desirable change in pH is 0.3. Intervals between adding titrant doses were 2–5 min and the amount of added portions was 0.05–0.1 mL. The duration of forward and backward runs (titration with base and acid titrant, respectively) altogether was about 85 min. The net proton surface excess amount (Δn*^σ^*_H+,OH−_, mol/g) is the difference of H^+^ and OH^−^ surface excess amounts (n*^σ^*_H+_, n*^σ^*_OH−_) related to unit mass of solid (Δn*^σ^*_H+,OH−_= n*^σ^*_H+_ − n*^σ^*_OH−_). The surface excess amount defined for adsorption [62] can be determined directly from the initial (c_i_^0^, mol/L) and equilibrium (c_i_^e^, mol/L) concentration of a given solute (n*^σ^*_i_ = (c_i_^0^ − c_i_^e^)V/m, where V is the volume (L) of liquid phase and m is the mass of adsorbent) in a dilute solution. The values n*^σ^*_H+_ and n*^σ^*_OH−_ were calculated at each point of titration using the actual activity coefficient from the slope of H^+^/OH^−^ activity vs. concentration straight lines for background electrolyte titration.

#### 3.2.3. Calorimetric Titration

To determine the heat effects of the acid-base titration of titania samples an isothermal microcalorimeter (TAM 2277, thermal activity monitor, Thermometric) was used at 25 °C. Only the washed TiO_2_ P25 sample was tested in the calorimetric experiments to eliminate uncontrolled acid-base reactions with significant heat effects. The samples contained 0.2 g titania powder dispersed in 10 mL electrolyte solution (0.005, 0.05, and 0.5 M KNO_3_, respectively) and were purged with argon to eliminate dissolved CO_2_ impurities. The suspensions were titrated separately with the portions of standard acid or base solutions under a CO_2_-free argon atmosphere. Blank titrations were also performed at the same concentrations of indifferent electrolyte KNO_3_ in the absence of the titania adsorbent. The heat flow across the titration cell of TAM was continuously recorded. The accuracy of the measured reaction heat data was tested by measuring the standard heat of reaction between THAM ((HO-CH_2_)_3_-C-NH_2_) and HCl (ΔH = −55 ± 1 kJ/mol). The combination of simultaneous chemical reactions and mixing processes yielded the overall heat flow (Q_meas_) in the calorimeter cell:

(21) $$Q_{meas}=Q_{ri}+Q_{mix}=\sum_{i}∆H_{ri}∆\xi_{i}+Q_{mix}$$

where ΔH_ri_ is the enthalpy change of reaction i, Δξ_i_ is the change of the extent of reaction i and Q_mix_ is the mixing heat of titrant’s portion. Considering acid-base titration, the following reactions can be accounted for: reactions with added acid (ΔH_a_), reactions with added base (ΔH_b_) and water formation (neutralization) reaction (ΔH_n_). Blank experiments were used to determine heat of mixing.

## 4. Conclusions

The pH and ionic strength-dependent surface charging of titanium oxide can be quantitatively characterized by potentiometric titration with acid or base in the presence of electrolytes. This method is sensitive to any acid/base-consuming processes other than surface protolytic reactions. Therefore, titration should be performed in a pH range where dissolution of oxide is negligible, and the sample should be purified from any acidic or alkaline contaminants. The acidic impurity of TiO_2_ P25 sample can be effectively removed by alkaline, then acidic treatments, and finally washing. Heat treatment at 400 and 600 °C, however, proved inefficient. Due to the anatase-to-rutile phase transition likely occurring at 600 °C, the active site density on the titania surface decreased after heating, potentially influencing the photocatalytic activity of titania, which is often calcined at elevated temperatures. This reduction in active sites may negatively affect the efficiency of photocatalytic reactions.

We conclude that it is essential to focus on: (i) precise work involving both the pH and the concentration calibration, allowing for concentration-based calculation, which is a prerequisite for obtaining correct values, (ii) the importance of purified materials (UP water, Cl-free TiO_2_, CO_2_-free titrants and conditions), and (iii) the limitations, such as the dissolution-free pH range of solid. These aspects are even more crucial during calorimetric titrations. The experimental data of heat-treated and washed samples were fitted using FITEQL software and applying the CC and DL models for the 2-pK surface protonation and deprotonation reactions. The fitting results were reasonable and consistent with literature data. Furthermore, the capacitance values calculated as fitting parameters of the CC model harmonized surprisingly well with experimental capacitances of TiO_2_ nanoparticles in the literature. An interesting observation is that SCM fitting from titration data based on accurate proton analytics resulted in correct surface charging parameters, even though the sample contained acid impurity. The quantitative characterization of surface charging for TiO_2_ P25 provides correct data for further modeling, such as photocatalytic reactions or adsorption of biomolecules at oxide/water interface, making our data useful for interpreting results obtained with P25 and for thermodynamic models. Using the CC model parameters, we simulated the protolytic reactions taking place in the calorimetric cell during acid-base titration steps. The derived enthalpies for the 2-pK surface charging reactions of titania were opposite in sign. We also confirmed that the partial molar enthalpies of these two surface reactions show weak ionic strength dependence, similar to findings for alumina [29]. This supports the theoretical prediction of Hall [28], who expected a systematic but negligible effect from the charge screening of electrolytes. By applying the thermochemical considerations of Hess’s law to our results for the 2-pK surface reactions, we derived enthalpies for the 1-pK model of surface protonation. Consistent application of thermochemical rules revealed that the seemingly contradictory results in literature arise from the formal difference between the reaction in the calorimetric cell and its interpretation. We found that the differences in reported molar enthalpies [21,22,23,34] are primarily due to the different experimental approaches and interpretations of the investigated surface charging reactions, aside from experimental error. The contradiction between positive and negative enthalpy values can also be resolved if the 1-pK protonation reaction in Equation (4) is used as the basis. In doing so, the enthalpies from the above works align well with each other and with the results of the present work. In conclusion, the thermodynamic parameters of titania from different sources are consistent within the experimental error of the applied methods. This study resolves a fundamental debate about the 1-pK and 2-pK approaches of the protolytic reactions at electrified interface of TiO_2_. The thermodynamic characterization of surface charging for TiO_2_ P25 in this work provides accurate data for thermodynamic databases, which is a key outcome.

## Figures and Tables

**Table 1:** Intrinsic equilibrium constants and fitting parameters for charge formation reactions (Equations (1) and (2)) occurring on the surface hydroxyl groups of titanium oxide P25 treated at 400 °C in electrolyte solutions.

	Electrolyte, KNO_3_			
	0.005 M	0.05 M	0.5 M	Average
Constant Capacitance Model (CCM)				
log *K*_a1_^int^(≡Ti-OH_2_^+^)	4.42	4.55	4.67	4.55 ± 0.12
log *K*_a2_^int^(≡Ti-O^−^)	−8.25	−8.55	−8.51	−8.44 ± 0.16
pH_PZC_	6.34	6.55	6.59	6.49 ± 0.14
T (≡Ti-OH)/M	0.003	0.012	0.045	
T (impurity)_fixed_/M	0.000418	0.000418	0.000418	
C_1/_F m^−2^	0.80	0.89	0.95	
WSOS/DF	4.76	8.62	6.06	
Diffuse Layer Model (DLM)				
log *K*_a1_^int^(≡Ti-OH_2_^+^)	5.25	5.72	6.45	5.80 ± 0.61
log *K*_a2_^int^(≡Ti-O^−^)	−7.44	−7.39	−6.74	−7.19 ± 0.39
pH_PZC_	6.34	6.55	6.60	6.50 ± 0.13
T (≡Ti-OH)/M	0.001	0.003	0.003	
T (impurity)_fixed_/M	0.000418	0.000418	0.000418	
WSOS/DF	2.00	5.33	6.23	

**Table 2:** Intrinsic equilibrium constants and fitting parameters for charge formation reactions (Equations (1) and (2)) occurring on the surface hydroxyl groups of titanium oxide P25 washed in electrolyte solutions.

	Electrolyte, KNO_3_			
	0.005 M	0.05 M	0.5 M	Average
Constant Capacitance Model (CCM)				
log *K*_a1_^int^(≡Ti-OH_2_^+^)	4.47	4.57	4.53	4.52 ± 0.05
log *K*_a2_^int^(≡Ti-O^−^)	−8.38	−8.35	−8.48	−8.4 ± 0.07
pH_PZC_	6.43	6.46	6.51	6.46 ± 0.04
T (≡Ti-OH)/M	0.0034	0.0120	0.0318	
T (impurity)_fixed_/M	0	0	0	
C_1/_F m^−2^	0.76	0.80	0.94	
WSOS/DF	13.90	12.70	3.91	
Diffuse Layer Model (DLM)				
log *K*_a1_^int^(≡Ti-OH_2_^+^)	5.25	5.86	5.99	5.70 ± 0.39
log *K*_a2_^int^(≡Ti-O^−^)	−7.60	−7.06	−7.02	−7.22 ± 0.32
pH_PZC_	6.42	6.46	6.51	6.46 ± 0.04
T (≡Ti-OH)/M	0.0014	0.0020	0.0028	
T (impurity)_fixed_/M	0	0	0	
WSOS/DF	7.57	10.58	4.49	

**Table 3:** Partial molar enthalpy values of the surface protolytic reactions in indifferent electrolyte solutions of TiO_2_ P25 at 25 °C.

Surface Reaction	≡Ti-OH+H^+^ ⇔ ≡Ti-OH_2_^+^	≡Ti-OH ⇔ ≡Ti-O^−^ + H^+^	≡Ti-OH^1/2−^ + H^+^ ⇔ ≡Ti-OH_2_^1/2+^
	Exothermic	Endothermic	Exothermic
*c*_KNO3_ (M)	Δ*H*_pr_ (kJ/mol)	Δ*H*_depr_ (kJ/mol)	(Δ*H*_pr_ − Δ*H*_depr_)/2 (kJ/mol)
0.005	−17.47	32.53	−25.00
0.05	−17.05	30.98	−24.01
0.5	−16.10	27.08	−21.59
average			−23.54 ± 1.75

## Data Availability

The raw data supporting the conclusions of this article will be made available by the authors on request.
